# Co-infection, super-infection and viral interference in HIV

**DOI:** 10.1186/1742-4690-10-S1-P72

**Published:** 2013-09-19

**Authors:** Azaria Remion, Marc Delord, Sentob Saragosti, Fabrizio Mammano

**Affiliations:** 1 INSERM U941, Université Paris Diderot, Paris, France; 2 Hôpital Saint-Louis, Paris, France

## Background

Viral interference is a phenomenon by which a virus-infected cell displays reduced susceptibility to re-infection. This phenomenon, also called superinfection resistance, is generally due to occupation or down-modulation of cellular receptors. In this respect, the principal mechanism of HIV superinfection resistance is down-modulation of the CD4 receptor from the cell surface mediated by Env, Vpu and Nef. Recent data, however, strongly suggest that HIV superinfection resistance also involves CD4-independent mechanisms that remain to be fully understood. Our aim was to explore the dynamics and mechanisms of CD4-independent viral interference in HIV.

## Material and methods

To quantify the infection by the first and the super-infecting virus, we used two isogenic HIV proviral clones, differing only in the expression of a marker protein (GFP and HSA). To analyze the dynamics of viral interference, the superinfecting virus was added at different times after the initial virus (0, 3, 6, 12, 18 and 24h). We prevented virus spread in culture by using single cycle virus infection. The number of cells expressing none, one, or both reporter proteins were measured by FACS analysis, and used to determine whether double infection occurred at frequencies expected for independent infection events.

## Results

In the case of co-infection (two viruses added at the same time), the frequency of double infected cells was significantly higher than it would be expected if the two infection events were independent. This result is consistent with previous literature [1].

By delaying the infection of the second virus (super-infection) and comparing the frequency of double infected cells to those observed for co-infection, we show evidence of viral interference starting 6h post-initial infection, which gained statistical significance when the second infection was performed 18h and 24h after the first one. Using a virus pseudotyped by the amphotropic-MLV Env for the second infection, we measured a similar level of interference up to 18h post-initial infection. This result strongly suggests that, for these early time points, virus interference is a CD4-independent phenomenon.

## Conclusions

Our experimental system allowed us to explore the efficiency of coinfection and the kinetics of viral interference in HIV. We confirmed that co-infection leads to a higher frequency of double infected cells than would be expected for independent infection events. In the setting of super-infection, only a few hours are required for the induction of measurable viral interference. This rapid phenomenon is largely independent of CD4-expression. Our experimental system will allow the exploration of the viral genes and the cellular mechanisms responsible for the early establishment of HIV interference.

## References

[B1] Dang Nonrandom HIV-1 infection and double infection via direct and cell-mediated pathways Proc Natl Acad Sci U S A 2004 101 632 637 10.1073/pnas.0307636100 14707263 PMC327199

